# Outpatient Management of Patients With Angina With No Obstructive Coronary Arteries: How to Come to a Proper Diagnosis and Therapy

**DOI:** 10.3389/fcvm.2021.716319

**Published:** 2021-11-02

**Authors:** Joan G. Meeder, Mariëlle J. Hartzema-Meijer, Tijn P. J. Jansen, Regina E. Konst, Peter Damman, Suzette E. Elias-Smale

**Affiliations:** ^1^Department of Cardiology, VieCuri Medical Center, Venlo, Netherlands; ^2^Department of Cardiology, Radboud University Medical Center, Nijmegen, Netherlands

**Keywords:** coronary vascular dysfunction, angina pectoris, ANOCA, INOCA, microvascular, vasospasm, vasomotor disorders

## Abstract

Two-thirds of women and one-third of men who undergo a clinically indicated coronary angiography for stable angina, have no obstructive coronary artery disease (CAD). Coronary vascular dysfunction is a highly prevalent underlying cause of angina in these so called “Angina with No Obstructive Coronary Arteries (ANOCA)” patients, foremost in middle aged women. Coronary vascular dysfunction encompasses various endotypes, namely epicardial and microvascular coronary spasms, impaired vasodilatation, and increased microvascular resistance. ANOCA patients, especially those with underlying coronary vascular dysfunction, have an adverse cardiovascular prognosis, poor physical functioning, and a reduced quality of life. Since standard ischemia detection tests and coronary angiograms are not designed to diagnose coronary vascular dysfunction, this ischemic heart disease is often overlooked and hence undertreated. But adequate diagnosis is vital, so that treatment can be started to reduce symptoms, reduce healthcare costs and improve quality of life and cardiovascular prognosis. The purpose of this review is to give a contemporary overview of ANOCA with focus on coronary vascular dysfunction. We will provide a possible work-up of patients suspected of coronary vascular dysfunction in the outpatient clinical setting, based on the latest scientific insights and international consensus documents. We will discuss the value of ischemia detection testing, and non-invasive and invasive methods to diagnose coronary vascular dysfunction. Furthermore, we will go into pharmacological and non-pharmacological therapeutic options including anti-anginal regimens and lifestyle interventions.

## Introduction

Angina pectoris affects more than 100 million people worldwide, and is the most common symptom of myocardial ischemia (1). Two-thirds of women and one-third of men who undergo a coronary angiogram (CAG) for symptoms of cardiac ischemia do not have obstructive coronary artery disease (CAD) (2–6). In the majority (59–89%) of these so called ANOCA (Angina with No Obstructive Coronary Arteries) patients, the symptoms are caused by coronary vascular dysfunction (7, 8), comprising epicardial vasospasm and/or coronary microvascular dysfunction (CMD) that includes microvascular coronary spasm, increased microvascular resistance and/or decreased vasodilatory capacity as measured by coronary flow reserve (CFR) (9, 10). This condition is more common in women than in men. A recent study in 1,379 patients with ANOCA showed coronary artery dysfunction in 70% of women vs. 43% of men (8). Women are more likely to have coronary vascular dysfunction without a history of obstructive CAD, while men with this condition are more likely to have a history of coronary revascularization (11). Because standard diagnostic tests for anginal symptoms are aimed at evaluating obstructive CAD, coronary vascular dysfunction is often overlooked. Accruing evidence shows that coronary vascular dysfunction can be properly diagnosed with techniques such as an invasive coronary vasomotor test and that treatment based on the results of such a test leads to reduction of symptoms and improvement of quality of life (12). This review is intended to give tips and tricks to adequately recognize, diagnose and treat patients with (suspected) coronary vascular dysfunction in the outpatient clinical setting.

## Why is it Important to Identify Patients With Coronary Vascular Dysfunction?

Patients with symptoms and signs suggestive of myocardial ischemia without obstructive CAD have an increased cardiovascular risk compared to persons without anginal symptoms. In 917 female patients with chest pain, the composite endpoint of myocardial infarction or cardiovascular death after 10 years of follow-up occurred in 6.7% of women without coronary artery disease, 12.8% of women with non-obstructive CAD, and 25.9% of women with obstructive CAD, respectively (13). A meta-analysis of more than 35,000 patients with ANOCA confirmed the increased risk of myocardial infarction and death. A worse prognosis was seen in patients with proven ischemia vs. no ischemia and patients with non-obstructive coronary disease vs. “normal” coronaries (14). Patients with proven coronary vascular dysfunction also have an increased risk of myocardial infarction, cerebral infarction, heart failure and (cardiovascular) death (15, 16). In addition to a worse cardiovascular prognosis, the disease is often associated with persistent symptoms, leading to unnecessarily repeated diagnostic examinations targeting obstructive CAD, first aid visits, hospital admissions, a reduced quality of life, and reduced labor participation (4, 14, 17). There are several treatment options for coronary vascular dysfunction. A recent randomized trial comprising 150 ANOCA patients, the Stratified Medical Therapy Using Invasive Coronary Function Testing in Angina (CorMiCa) trial, shows that targeted treatment after demonstration of coronary vascular dysfunction significantly reduces anginal complaints and improves quality of life (12). Treatment options focus on symptom relief, improvement of the cardiovascular risk profile, quality of life, and prognosis. Timely recognition of this disease, and thus the start of treatment, is therefore essential.

## Underlying Coronary Pathophysiology

The coronary microcirculation is an adaptive system that regulates myocardial perfusion (18). While the epicardial coronaries (diameter > 400 μm) act as transport vessels, the pre-arterioles and arterioles in the coronary microcirculation mainly determine resistance. The autoregulation of this resistance makes it possible to maintain a constant blood flow over a wide range of coronary perfusion pressures, delivering oxygen and nutrients to the tissue and removing waste products. Several mechanisms play a role in this autoregulation, namely: myogenic regulation by the vascular smooth muscle cells, metabolic control by metabolites from adjacent myocardial cells, endothelial function responsive to changes in vascular wall tension, autonomic innervation, and circulating hormones including endothelium dependent relaxation factors such as nitric oxide (NO) and prostaglandins (18, 19) and vasoconstrictor agents such as histamine, norepinephrine, and serotonin (20).

Coronary vascular dysfunction can be caused by functional and structural abnormalities. Functional abnormalities include endothelial and non-endothelial related pathology (21). Endothelial function can be tested by evaluating the response of the coronary arteries to acetylcholine. If coronary vasodilation occurs after administration of acetylcholine, this indicates well-functioning endothelium. When vasoconstriction occurs, it indicates endothelial dysfunction. The latter is often found in the early phase of atherosclerosis (18, 22). Non-endothelial mediated functional abnormalities are related to decreased diastolic time, increased intramyocardial pressure, increased intracavitary pressure, and/or tissue edema. Structural abnormalities include microvascular remodeling in arterioles (intimal thickening, smooth muscle cell proliferation and perivascular fibrosis) and decreased capillary density resulting in increased microvascular resistance. This is found, among other things, in left ventricular hypertrophy, calcium surplus and amyloidosis (21). In general. any dysfunction based on non-endothelial mediated functional and/or structural abnormalities of the coronary microcirculation can be demonstrated by CFR and microvascular resistance measurements.

## Clinical Presentation

Coronary vascular dysfunction includes CMD and/or epicardial spasm. Since both conditions may differ in clinical symptoms and risk factors, the following sections differentiate between them where relevant.

### Symptoms

Angina pectoris is an important symptom of coronary vascular dysfunction. Angina equivalents such as exertional dyspnea and fatigue may also be an expression of this condition. Microvascular angina (MVA)—due to CMD—is difficult to distinguish from classic angina due to obstructive CAD because both are often exercise-related. MVA is more likely if the angina continues after exercise cessation, starts after exercise and/or has limited response to nitroglycerin administration (23). In addition, MVA is more often triggered by palpitations or mental stress (24). The intensity of symptoms can vary over time and can be so severe that patients are limited in daily life activities. Angina at rest often occur in addition to exercise-related complaints (25). This is likely due to a vasospastic component of coronary vascular dysfunction (26). Pure vasospastic (Prinzmetal) angina usually occurs at rest, mainly at night and/or early morning. However, exercise-related symptoms can also be due to vasospasm (27, 28). Based on symptoms only, it is generally not possible to make a good distinction between coronary vascular dysfunction and obstructive CAD. In clinical practice, a significant obstruction must therefore be ruled out by a CAG or coronary computed tomography (CT) scan.

### Risk Factors

#### Coronary Microvascular Dysfunction

Classic cardiovascular risk factors such as age, hypertension, diabetes, smoking, and especially dyslipidemia and obesity are also involved in CMD (29). But these risk factors only explain part of the occurrence of this disease (30). Specific risk factors for CMD (Table 1) include (premenopausal) migraine, rheumatic diseases or inflammatory bowel diseases (31–33). Female-specific risk factors, such as pre-eclampsia, Hemolysis Elevated Liver enzymes and Low Platelets (HELLP) syndrome, gestational hypertension and diabetes, recurrent spontaneous abortion, and menopausal status, may also play a role (34). It is therefore important to not only concentrate on classical risk factors, but also on migraine, inflammatory disease and female specific risk factors when asking the patient his or her medical history.

**Table 1:** Non-classical risk factors and triggers playing a role in ANOCA.

**Non-classical risk factors for CMD**	**Triggers for epicardial spasm**
(premenopausal) Migraine	Alcohol (especially withdrawal)
Rheumatic diseases	Smoking
Inflammatory bowel diseases	Hyperventilation
Pre-eclampsia/HELLP syndrome	Mental stress
Gestational hypertension and diabetes	Drug use (cocaine, amphetamines)
Recurrent spontaneous abortion	Allergic reaction
Menopausal status	Exposure to cold
	Vasoconstrictor agents (e.g., propranolol, anti-migraine medication)
	Chemotherapy

#### Epicardial Vasospasm

With the exception of smoking, classical risk factors such as hypertension, dyslipidemia and diabetes are not clearly related to vasospastic angina. Smoking is not only a risk factor but can also trigger vasospasm attacks (9). Anginal episodes can also be triggered by (withdrawal of) alcohol, hyperventilation, mental stress and daily hassles, stimulant drug use (e.g., cocaine, amphetamines), allergic reactions, exposure to cold (9, 35–39) and vasoconstrictive medications (e.g., propranolol, anti-migraine medication) and chemotherapy (Table 1) (39–41). Vasospastic angina is likely related to other vasospastic conditions such as migraine and Raynaud's phenomenon (42, 43).

## Diagnosis of Coronary Vascular Dysfunction

In the diagnostic work-up of angina (equivalents), in patients with and without a history of obstructive CAD, obstructive CAD as underlying cause of symptoms will have to be ruled out by coronary CT or CAG before considering coronary vascular dysfunction. Consideration should also be given to possible alternative diagnoses.

### Alternative Diagnoses

Chest pain without obstructive CAD can be due to other conditions than coronary vascular dysfunction. Differential diagnoses can be divided into three groups: (1) non-cardiac, (2) cardiac non-ischemic, and (3) cardiac ischemic (44, 45). There are multiple causes of non-cardiac chest pain, including gastrointestinal causes such as gastroesophageal reflux, pulmonary disorders like asthma, musculoskeletal complaints like costochondritis, and psychiatric causes such as anxiety/panic disorders (46, 47). Cardiac non-ischemic pain can be the result of pericarditis, increased intraventricular pressure in systolic or diastolic heart failure or valvular heart disease or altered pain perception (nociception) (48). Cardiac ischemic etiologies include, in addition to coronary vascular dysfunction, myocardial bridging (49). Hypertension deserves special attention. Hypertensive patients often experience chest pain despite the absence of obstructive CAD (50). Strict treatment of blood pressure (target tension 120/70 mmHg) often improves symptoms of chest pain and/or exertional dyspnea.

## Diagnostic Criteria for Coronary Vascular Dysfunction (CMD and Epicardial Spasm)

In 2017, diagnostic criteria for epicardial spasms were published by the international Coronary Vasomotor Disorders (COVADIS) study group (Table 2) (9).

**Table 2:** Diagnostic criteria for epicardial spasm.

**(1) Nitrate-responsive angina**—during spontaneous episode, with at least one of the following:
(a) Rest angina—especially between night and early morning
(b) Marked diurnal variation in exercise tolerance—reduced in morning
(c) Hyperventilation can precipitate an episode
(d) Calcium channel blockers (but not β-blockers) suppress episodes
**(2) Transient ischemic ECG changes**—during spontaneous episode, including any of the following in at least two contiguous leads:
(a) ST segment elevation ≥ 0.1 mV
(b) ST segment depression ≥ 0.1 mV
(c) New negative U waves
**(3) Coronary artery spasm**—defined as transient total or subtotal coronary artery occlusion (>90% constriction) with angina and ischemic ECG changes either spontaneously or in response to a provocative stimulus (typically acetylcholine, ergonovine, or hyperventilation)

In 2018, this group drew up diagnostic criteria for CMD (Table 3) (10).

**Table 3:** Diagnostic criteria for coronary microvascular dysfunction (CMD).

**1. Symptoms of myocardial ischemia**
a) Effort and/or rest angina
b) Angina equivalents (i.e., shortness of breath)
**2. Absence of obstructive CAD (<50% diameter reduction or FFR by >0.80) by:**
a) Coronary CTA
b) Invasive coronary angiography
**3. Objective evidence of myocardial ischemia**
a) Ischemic ECG changes during an episode of chest pain
b) Stress-induced chest pain and/or ischemic ECG changes in the presence or absence of transient/reversible abnormal myocardial perfusion and/or wall motion abnormality
**4. Evidence of impaired coronary microvascular function**
a) Impaired coronary flow reserve (cut-off values depending on methodology used: ≤2.0 and ≤2.5)
b) Abnormal coronary microvascular resistance indices (e.g., IMR > 25)
c) Coronary microvascular spasm, defined as reproduction of symptoms, ischemic ECG shifts but no epicardial spasm during acetylcholine testing.
d) Coronary slow flow phenomenon, defined as TIMI frame count >25

## The Value of Non-invasive Ischemia Detection

If 3 of the 4 COVADIS criteria are met (Table 3), a diagnosis of CMD is likely. If all criteria are met, a definite diagnosis of CMD can be made. The gold standard to diagnose coronary vascular dysfunction is an invasive coronary vasomotor test that can comprehensively test all 4 endotypes of coronary vascular function: epicardial and microvascular coronary vasospasms, vasodilatory capacity and microvascular resistance. As can be appreciated in Table 3, having symptoms without obstructive CAD together with objective ischemia leads to a likely diagnosis of CMD. However, “objective ischemia” is a broad concept in the criteria, and it should be noted that several studies have shown no good correlation between demonstrated ischemia by a non-invasive ischemia detection test [exercise testing, stress Cardiac Magnetic Resonance (CMR), Single-Photon Emission Computed Tomography (SPECT), stress echocardiogram] and invasively determined coronary vascular dysfunction. This could be related to the fact that coronary vascular dysfunction causes a heterogeneous pattern of non-transmural ischemia, which is not visible as a regional perfusion defect (51, 52). This has the important implication that a negative ischemia detection test does not rule out coronary vascular dysfunction. Currently, the invasive coronary vasomotor test is very limited available, and therefore not feasible in the majority of patients. Although currently, a clear diagnostic work-up is lacking, in our opinion, it is worthwhile to do a non-invasive stress test in patients for which this test is not accessible, especially in patients with exercise-related symptoms. If this ischemia detection test is positive, the suspicion of coronary vascular dysfunction is reinforced. The stress test can be a “simple” exercise test with the advantage that the effort is physiological, and the test is readily available and cheap.

## Demonstration of Coronary Vascular Dysfunction

As mentioned above, the gold standard to diagnose coronary vascular dysfunction, the invasive coronary vasomotor test, is currently scarce. Hence, for outpatient clinical management it is important to know the value of non-invasive alternatives that can be used to evaluate of coronary vascular dysfunction.

### Non-invasive Diagnostic Options for Coronary Vascular Dysfunction

CFR can be measured non-invasively with various imaging techniques. All methods evaluate coronary flow (velocity) or perfusion during hyperemia using adenosine vs. rest.

Cardiac Positron Emission Tomography (PET), a radionuclide imaging technique, is considered the most reliable method using ^15^O-water, ^13^N-ammonia, or ^82^rubidium tracers (53). It has been well validated for accurate and reproducible quantification of regional myocardial blood flow (MBF) and CFR in the myocardium (54, 55). PET is considered the gold standard for the non-invasive assessment of CFR and correlates well with invasive assessment of CFR (56). A CFR < 2 is diagnostic for ischemia and thus CMD and related to a worse cardiovascular prognosis (53, 57). Despite, PET is not widely used due to some major limitations, namely, high expense, the necessity of an on-site cyclotron when using ^15^O-water and ^13^N-ammonia, and the involvement of radiation (58–60).

In CMR, a technique has been developed to determine the Myocardial Perfusion Reserve Index (MPRI). Using a contrast medium (gadolinium), diffusing from the microvasculature into the interstitial space, perfusion signal intensity upslopes are evaluated in stress (induced with adenosine) vs. rest, the ratio being the MPRI, which is considered a surrogate for the CFR (61). CMR is more widely available than PET, less expensive and involves no radiation However, further validation studies of MPRI in relation to the results of coronary vasomotor testing are needed before this technique is ready to be clinically used.

With TransThoracic Doppler Echocardiography (TTDE), the Coronary Flow Velocity Reserve (CFVR) can be determined in the Left Anterior Descending coronary (LAD). The CFVR is the ratio of the peak velocity in hyperemia (using systemic adenosine) vs. the peak velocity in rest in the LAD and a surrogate for CFR. A CFR < 2.5 is considered diagnostic for CMD. Although echocardiography is readily available and inexpensive, this method has limited application because it requires expertise from the echocardiographer and not all patients have a suitable ultrasound window (62, 63).

PET, CMR and TTDE have an ESC IIb recommendation (i.e., may be considered) for the detection of coronary vascular dysfunction (64). However, the described methods only assess CFR, which is reflecting just 1 of the 4 endotypes of coronary vascular dysfunction. Although research is being done on non-invasive measures of microvascular resistance, (65) currently, this endotype cannot be assessed non-invasively in clinical practice. Furthermore, vasospastic disease cannot be evaluated adequately by the contemporary non-invasive techniques such as cold-pressor PET. It cannot distinguish between epicardial and microvascular vasospasm and, most essential, does not correlate well with invasively assessed vasospasm (15). This is an important limitation since vasospasm is the most prevalent endotype in patients with coronary vascular dysfunction, occurring in 81–97% of patients diagnosed with coronary vascular dysfunction, while an abnormal CFR or microvascular resistance without vasospasms occurred in only 3–19% (52, 66, 67). Therefore, one should realize that the diagnosis of coronary vascular dysfunction is easily missed with non-invasive diagnostics.

### The Invasive Coronary Vasomotor Test

As mentioned above, the coronary vasomotor test is currently the only test that can comprehensively evaluate all endotypes of coronary vascular dysfunction. During a CAG, obstructive CAD is ruled out, after which vasomotor tests are performed.

**Figure 1:**
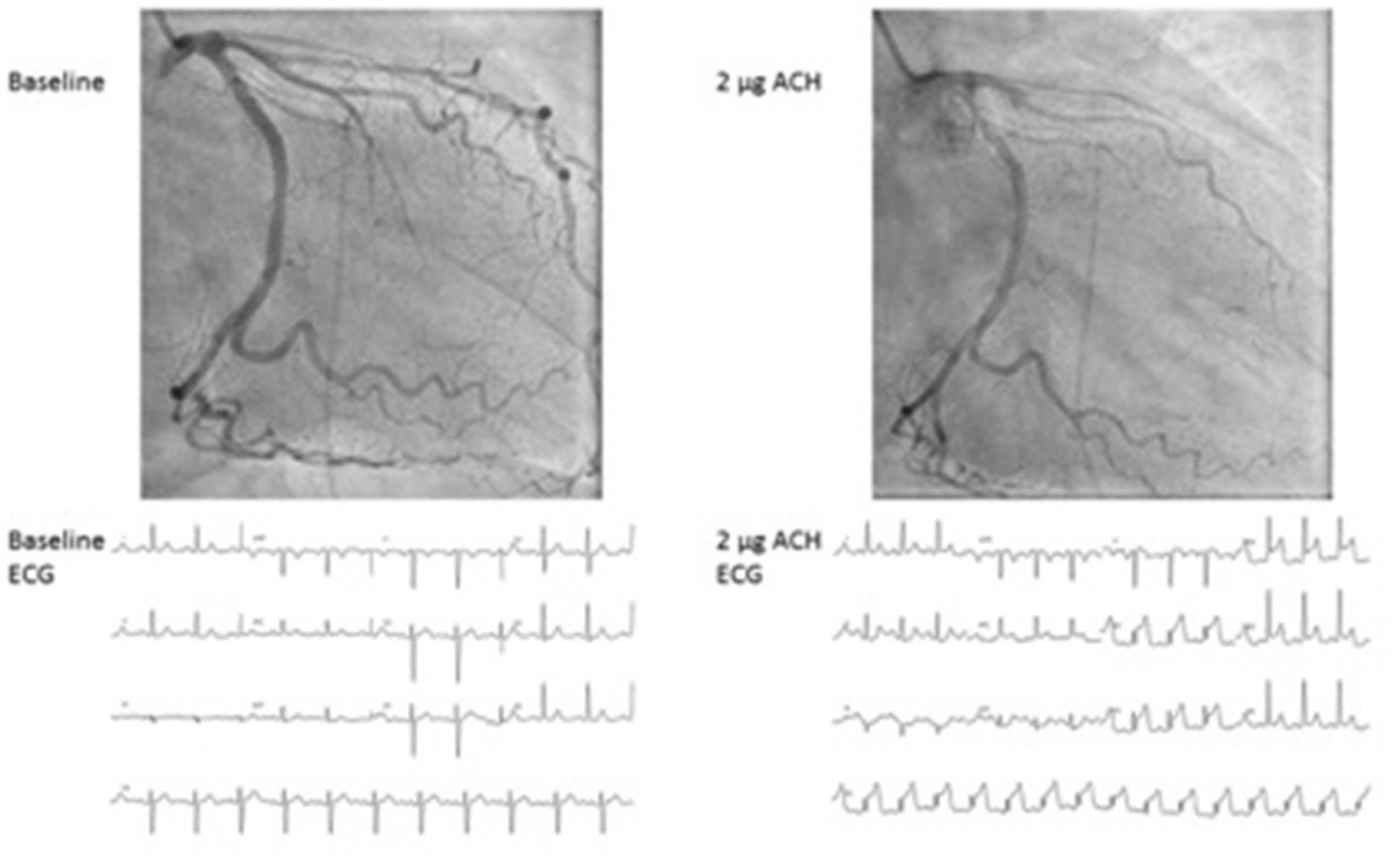
Epicardial spasm provoked by acetylcholine during coronary vasomotor test. Case of a 46-year-old female with severe chronic angina. At 2 mcg of acethylcholine a severe spasm of the LAD occurs with concurrent severe angina and ST elevation at the ECG. ACH, Acetylcholine; ECG, electrocardiogram.

To evaluate coronary vasospasm, ascending doses (usually 2, 20, 100, and 200 μg) of acetylcholine are given in the left coronary artery with continuous monitoring of symptoms and 12-channel ElectroCardioGram (ECG). An alternative to acetylcholine is ergonovine, but it is less effective, especially in women (68). The acetylcholine test, as mentioned in Tables 2, 3, is positive for epicardial spasms if recognizable symptoms occur, accompanied by ischemic ECG changes and a ≥ 90% reduction of the coronary lumen: an example of a positive acetylcholine test for epicardial spasm can be appreciated in Figure 1. If there are recognizable symptoms and ischemic ECG changes, but < 90% lumen reduction, the diagnosis of microvascular spasm is made. The acetylcholine test ends with the administration of nitroglycerin to ensure that there is complete vasorelaxation of the coronaries and that the patient is free of complaints.

Using systemic infusion of adenosine, the coronary flow reserve (CFR) is determined, defined as the ratio between hyperemic coronary flow vs resting flow. This can be done by means of thermodilution or Doppler (56). An invasively measured CFR < 2 is generally used as a cut-off value for the detection of ischemia in CMD. The basis for this is the finding that CFR < 2, measured by PET, is related to a clearly worse cardiovascular prognosis (53, 57). CFR > 2.5 is considered normal. CFR values between 2 and 2.5 form a gray area. In the same session, the microvascular resistance is measured: if thermodilution is used, the index of microvascular resistance (IMR) is determined, if the evaluation is done with Doppler, the hyperemic microvascular resistance (HMR) is determined (69). IMR of 25 U is generally used as cut-off, with values above 25 being diagnostic for CMD (Table 3). For HMR, 2.5 mmHg/cm/s is often used as a cut-off value for the diagnosis of microvascular disease (57, 70).

As mentioned in the introduction, in the majority of patients with ANOCA, coronary vascular dysfunction is found on invasive coronary vasomotor test (59–89%) (7, 8). Most of the patients with coronary vascular dysfunction have an abnormal acetylcholine test (52, 66, 67). In addition, patients may have an abnormal CFR or microvascular resistance which might influence prognosis (67). Therefore, we recommend that in all patients undergoing an invasive test a complete coronary vasomotor test with administration of both acetylcholine and adenosine should be performed.

## Safety of the Invasive Coronary Vasomotor Test

Recent large studies have shown that coronary vasomotor tests can be performed safely. Complication risks of 0–0.7% are found for the occurrence of serious complications such as myocardial infarction, ventricular fibrillation or death. This is comparable to a CAG with FFR measurement (22, 71).

## Proposed Diagnostic Algorithm

**Figure 2:**
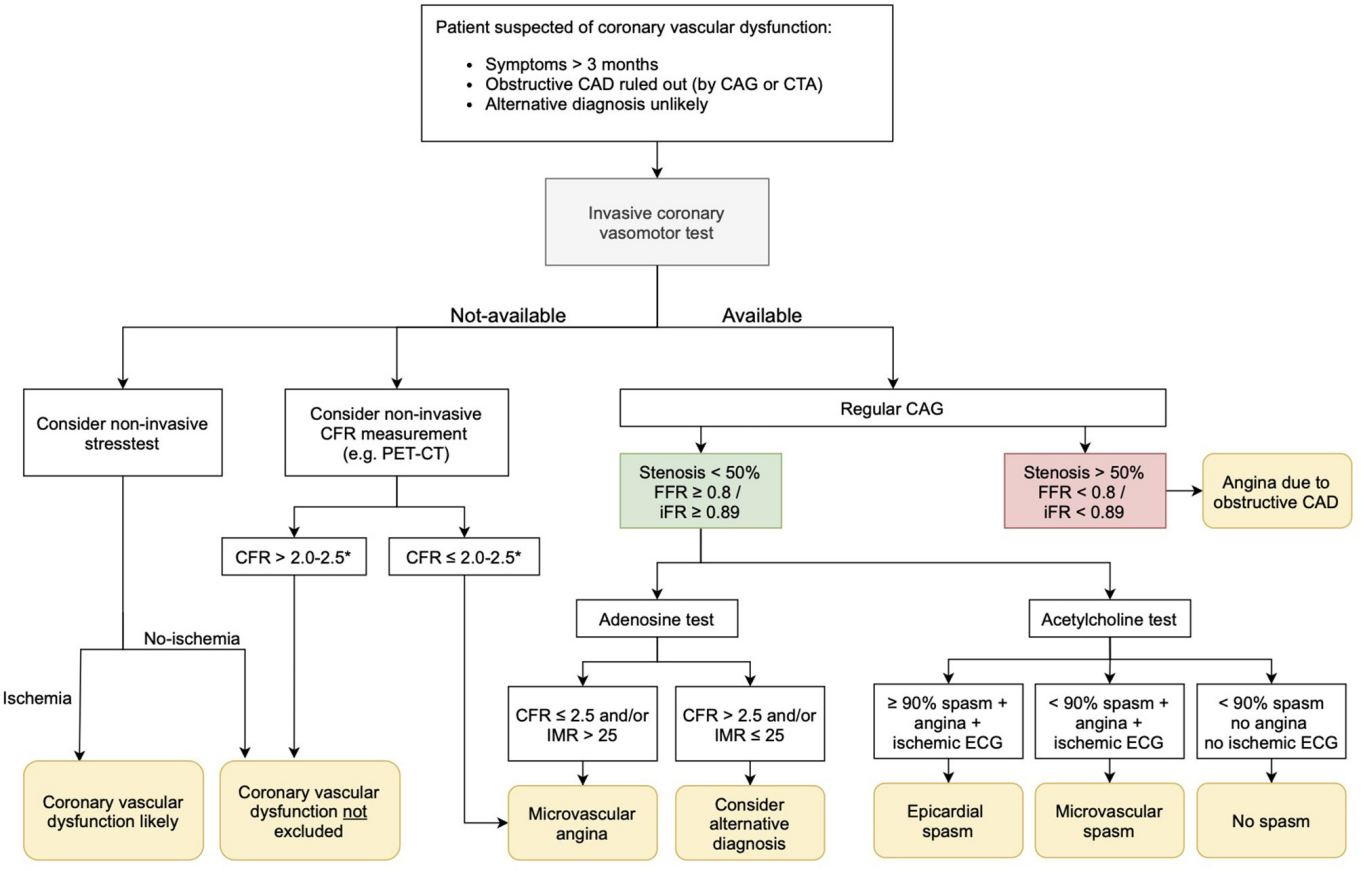
Possible diagnostic algorithm.

Coronary vascular dysfunction plays a substantial pathogenic role across the spectrum of ischemic heart disease including patients with no obstructive CAD and individuals with obstructive CAD, as well as those with persisting angina after anatomically successful coronary recanalization/revascularization. So, what to do in clinical practice with patients suspected of coronary vascular dysfunction? In current clinical practice, we still lack a generally used work-up for patients with ANOCA. In Figure 2, we propose a possible diagnostic algorithm. In patients with angina (equivalents) lasting at least 3 months, first of all, obstructive CAD as underlying cause of symptoms should be ruled out with a coronary CT scan or CAG. Also, alternative diagnoses should be considered, and if likely, ruled out. If an invasive coronary vasomotor test is available, the next step would be to perform such a test. We propose to do a comprehensive test using both acetylcholine and adenosine to investigate all endotypes of coronary vascular dysfunction to get a definite diagnosis, including the endotype of dysfunction on which treatment can be based (57). In patients who do not wish to undergo a vasomotor test or in centers where this test is not available, clinicians could consider a non-invasive stress test to evaluate ischemia. We would recommend a “simple” but physiological exercise test, especially in patients with exercise related symptoms. When this exercise test indicates cardiac ischemia, the diagnosis of coronary vascular dysfunction is strengthened. If it is negative for ischemia, the diagnosis vascular dysfunction cannot be ruled out. In hospitals with expertise in TTDE or PET, non-invasive evaluation of the CFR could be performed. If CFR is <2–2.5 (depending on the method used), CMD is diagnosed. If the CFR is normal, coronary vascular dysfunction is still not ruled out. So, non-invasive ischemia testing and CFR measurements can only be used to rule in, or strengthen the diagnosis, but cannot be used to rule out coronary vascular dysfunction.

If it is possible to refer patients for coronary vasomotor testing in another hospital, we advise that an invasive test should be considered in the following cases in particular: (1) If having a definite diagnosis is important to the patient, e.g., to promote acceptance of the disease or to have a clear diagnosis in case of a working dispute. (2) If having a definite diagnosis is important to the cardiologist, e.g., when medical therapy is not effective and doubts raise about the likelihood of the diagnosis.

We recommend to start treatment in all patients with suspected coronary vascular dysfunction and adjust it according to effectiveness, side effects, and, if possible, the definite diagnosis. Recommendations for therapy are given in the section below.

## Treatment Options

Recommendations for drug treatment of coronary vascular dysfunction are mainly based on small studies because large outcome trials are lacking to date (72). In the literature, recommendations exist for both CMD and epicardial spasm. Since the exact underlying mechanism is usually unknown in patients with suspected coronary vascular dysfunction, this distinction is not so strict in clinical practice. The choice of medication and dosage will differ per patient, not only based on the possible underlying pathophysiological mechanism, but also on parameters such as heart rate and blood pressure, effectiveness, and the occurrence of side effects.

## Cardiovascular Risk Management

Since classical cardiovascular risk factors also play a role in CMD and epicardial spasms, it is recommended that these be strictly controlled with statins, antihypertensive drugs, anti-diabetic therapy, and lifestyle modifications such as weight reduction and smoking cessation (23, 73, 74). It should be emphasized that it is important to control blood pressure very tightly (target tension 120/70 mmHg). This is not only important in the context of lowering the cardiovascular risk, but in practice it has been found that strict blood pressure regulation often provides clear complaints relief. Statins, in addition to their cholesterol-lowering effect (75), are recommended because of their beneficial effect on endothelial function (76). In addition, several studies have shown a beneficial effect of statins in the prevention of coronary spasm attacks (77). When epicardial spasm is suspected, triggers such as smoking/co-smoking and drug use (including cocaine or amphetamines) should be strongly discouraged (23). Angiotensin-Converting-Enzyme (ACE) inhibitors and angiotensin II antagonists are not only effective in lowering blood pressure, but also improve endothelial function and have a beneficial effect on both CMD and epicardial vasospasm (40, 78, 79).

## Aspirin

The role of aspirin in the treatment of CMD is unclear. The 2013 ESC guideline for stable angina pectoris recommends aspirin for all patients with microvascular angina pectoris (23). The current ESC guideline for chronic coronary syndromes (2019) does not provide clear guidance on this (74). Obviously, aspirin is indicated in patients with a previous cardiovascular event (80). However, for patients without a cardiovascular event, this recommendation is not based on scientific studies. In clinical practice, aspirin is given at the discretion of the treating cardiologist. Some experts in coronary vascular dysfunction do not give aspirin because 3 recent large clinical trials have shown that primary prevention with aspirin is not useful, even in high-risk populations (81). Others are more liberal in giving aspirin, especially in patients with evident but non-obstructive atherosclerosis on CAG, a high calcium score and/or non-calcified plaque on CT coronary angiography and/or a positive ischemia detection test. In epicardial coronary spasm, high-dose aspirin (as given for pericarditis) is not recommended because the blockade of prostacyclin production can aggravate the spasm (82). The use of low-dose aspirin (80–100 mg per day) in patients without concomitant obstructive CAD is still under discussion due to the lack of robust outcome data. Therefore, aspirin is not routinely given in patients with coronary spasm without obstructive CAD (77). Of course, one could deviate from this routine in individual cases, for example in patients with non-obstructive atherosclerosis with focal epicardial spasm at the location of coronary plaques.

## Anti-Anginal Treatment

As can be appreciated in Table 4, several anti-anginal treatment options are available for CMD and epicardial spasm.

**Table 4:** Anti-anginal treatment for CMD and epicardial spasm.

**Medication**	**Coronary microvascular** **dysfunction**	**Epicardial spasm**
First-line	Calcium channel blocker (Diltiazem, Nifedipine) ß-blocker (Nebivolol, Atenolol, Carvedilol, Labetalol) Short acting nitrate	Calcium channel blocker (Diltiazem, Nifedipine) Short acting nitrate
Second-line	Long acting nitrate Nicorandil Ranolazine	Long acting nitrate Nicorandil
Third-line	Imipramine Trimetazidine	

### CMD

#### First-Line Therapy

In patients with predominantly resting symptoms, calcium channel blockers are recommended because they have been shown to be effective in both epicardial spasms and CMD (40, 72, 83). Both non-dihydropiridines (e.g., diltiazem) and dihydropiridines (e.g., nifedipine retard) calcium antagonists can be given. β-blockers are recommended in patients with predominantly exercise-related complaints (23). In terms of choice of beta-blocker, atenolol and nebivolol are particularly recommended (40). Atenolol improves exercise capacity and anginal symptoms (84). Nebivolol is not only a selective β-1 receptor blocker, but also has vasodilatory effects through NO production, which is likely to be beneficial in case of a vasospastic component (85). Several small studies showed a better effect of nebivolol than metoprolol (86, 87). In addition to nebivolol, carvedilol and labetalol (β-blockers with both alpha-1 and β-adrenergic receptor antagonist properties) are recommended for CMD due to their vasodilatory effect (88). Short-acting nitrates are recommended for stopping attacks of angina, although this will not relieve symptoms in all patients (23, 40, 76).

#### Second-Line Therapy

In patients with refractory symptoms and/or intolerance to the first-line medication, other anti-anginal medications such as long-acting nitrates or ranolazine may be given (23). These can be used in addition to first-line therapy (40). In practice, nicorandil often works better than other long-acting nitrates because, in addition to its effect on nitric oxide production, it has a beneficial effect on the smooth muscle cells around the vessel wall (40, 89). Long-acting nitrates relieve symptoms in about half of patients with suspected CMD (23, 40, 90). Ranolazine is a sodium channel blocker that reduces intracellular calcium in cardiomyocytes leading to improved intraventricular relaxation, potentially improving microcirculation. Studies are unclear about the effect of this drug, although a recent randomized trial in 81 patients showed that ranolazine improves symptoms and myocardial perfusion in patients with a CFR < 2.5 (91).

#### Third-Line Therapy

In case of insufficient relief of symptoms on first- and second-line therapy, consideration should be given to establish or dismiss the diagnosis coronary vascular dysfunction with a coronary vasomotor test if this has not yet been done. Third-line medication includes Trimetazidine, an anti-ischemic metabolic agent that improves myocardial glucose utilization through inhibition of fatty acid metabolism. It improves angina and stress testing results when compared to conventional therapy (92). Also, a low dose of imipramine, a tricyclic antidepressant can be considered, acting as a pain reliever (40). Besides medical therapy, non-medical therapy, as will be discussed below, can be very helpful in patients with refractory symptoms.

### Epicardial Spasm

#### First-Line Therapy

First-line therapy consists of a (non-) dihydropyridine calcium channel blocker and a short-acting nitrate to stop vasospasm attacks (23, 74). Non-selective β-blockers, such as propranolol, should be avoided if there is (suspected) coronary artery spasm, as they can trigger spasm (40, 41). However, as mentioned above, nebivolol was shown to reduce coronary vasospasm, although not as effective as diltiazem (85).

#### Second-Line Therapy

If the effect on the complaints of first-line treatment is insufficient, a long-acting nitrate can be added (23). It can also be considered to combine a non-dihydropyridine with a dihydropyridine calcium antagonist, although this combination frequently causes side effects such as edema formation (93). Nicorandil has also been shown to be an effective agent for epicardial spasms and can be added if symptoms are insufficiently controlled (83).

## Experimental Therapy for Coronary Vascular Dysfunction

A number of novel drugs are promising, such as the rho kinase inhibitor Fasudil, which has been shown to be effective in preventing acetylcholine-induced vasospasm (94). Other potentially effective agents include type 3 and type 5 phosphodiesterase inhibitors (cilostazol and sildenafil, respectively). In a multicenter randomized trial of vasospastic angina patients refractory to amlodipine, cilostazol reduced angina frequency and intensity without serious adverse effects (95). In women with ANOCA, sildenafil acutely improved CFR among women with CFR <2.5 (i.e., CMD) (96). Endothelin 1 (ET-1) contributes to coronary endothelial dysfunction and its tonic inhibitory effect on myocardial perfusion is related to atherosclerosis risk factor burden (97). Two small randomized trials of an endothelin-1 (ET-1) receptor antagonist in MVA suggested a beneficial effect (98, 99). Currently, the Precision Medicine With Zibotentan in Microvascular Angina (PRIZE) trial is investigating the effect of zibotentan, an oral endothelin A receptor-selective antagonist, on symptoms, exercise duration and myocardial blood flow in patients with microvascular angina (ClinicalTrials.gov Identifier: NCT04097314). A comprehensive, contemporary overview of potential novel drugs is given by Bairey Merz et al. (92).

## Non-medical Therapy for Coronary Vascular Dysfunction

Non-medical anti-anginal therapy such as a Transcutaneous Electrical Nerve Stimulation) (TENS) can be considered for severe refractory symptoms (31). However, studies report varying results of this therapy on angina pectoris in CMD patients (100). Another option to relieve symptoms and improve quality of life is spinal cord stimulation, which modulates nociceptive signals and reduces ischemia through its anti-adrenergic effect. In addition, symptom relief has also been reported with “enhanced external counter pulsation (EECP),” a treatment in which inflatable cuffs are placed around the legs and buttocks which stimulate the return of blood to the heart through continuous inflation and deflation (101).

## Lifestyle Adjustments

Many patients with coronary vascular dysfunction are limited in their daily life by chronic, severe symptoms. Because it often concerns middle-aged women who work and/or have a family with growing children, the disease often has a major impact on daily life. There is little scientific literature available on the influence of lifestyle changes on symptoms. At the Radboud University Medical Center, Nijmegen, the Netherlands, we have over 5 years of experience with the guidance of these patients by a nurse practitioner. The following advice is based on this experience. Since the majority of cardiology clinics do not have a nurse practitioner, we have formulated the recommendations in such a way that they are generally applicable.

## Exercise

Exercise helps to reduce symptoms and improve exercise tolerance (102). A regular cardiac rehabilitation program is often too strenuous for patients with seriously debilitating symptoms. To date, no exercise program exists that is tailored to patients with ANOCA. We did in-depth interviews with 10 of our patients on this subject. Several important barriers to perform physical activity came up: anxiety to develop symptoms, mental pressure leading to symptoms and uncertainty of their physical limitations due to variation of symptoms over time. Regarding wishes for an exercise program, the patients found it important to exercise under supervision of a healthcare professional with knowledge of ANOCA, who knows how to train and give advice on coping with symptoms during or after physical activity, leading to a feeling of security and being taken care of. Next to fitness training they would like to train to perform their house holding activities,. The patients also stressed the importance of a minimum of stimuli (for example audio: the acoustics as well as the volume, preferably no music or loud voices) and pressure (for example setting goals with time limits) since it can trigger symptoms. Furthermore, they expressed the need for a slow start-up. Research has confirmed the importance to do a thorough warming-up (minimal 10 min on 50–60% of maximum intensity) to avoid “warm-up angina” (103, 104). Furthermore, experience learns that when patients exercise too intensively, it aggravates symptoms and often causes excessive fatigue the next day. Learning to listen to their body signals and take adequate actions, is difficult but essential to avoid these symptoms.

## Fatigue

Most patients suffer from fatigue as one of their symptoms. This is often a multifactorial problem occurring in coping with a chronic illness (105). An occupational therapist can offer practical help to make household chores easier to save energy by helping the patient pacing themself and enable energy conservation. Furthermore, they can give insight in the patients own activities and how to achieve a balanced lifestyle. With this insight, patients can make decisions on how to distribute their energy throughout the day/week and thereby limit the loss of functioning.

## Mental Stress

Mental stress and/or overstimulation can trigger symptoms, probably related to vasospasm (36). Patients with severe symptoms often report concentration problems and symptoms triggered by work deadlines or outside stimuli like traffic noise and social events. It is therefore important to teach patients how to deal with stress/outside stimuli. Possible interventions include mindfulness, yoga, Tai Chi, or walking in nature. A lot of patients also experience considerable mental stress during a disability trajectory. Occupational physicians or disability experts should therefore be well informed of the disease so that they can support the patient as good as possible. Another important cause of mental stress is having to deal with a chronic disabling disease at a relatively young age. Acceptance of the disease is very difficult for patients with severely limiting symptoms and can aggravate symptoms. Psychological counseling can offer help.

## Menopause Practitioner

If the symptoms appear to be related to the menopause, for example, anginal symptoms provoked by menopause-related increase in palpitations, it can be considered to refer patients to a menopause practitioner for additional tips and interventions.

## Knowledge Gaps and Future Directions in Outpatient Management

Although research on diagnostic and therapeutic strategies for coronary vascular dysfunction is rapidly expanding, many knowledge gaps still need to be filled. A prerequisite for improving healthcare for these patients is to increase awareness among cardiologists that coronary vascular dysfunction is a plausible cause underlying cardiac symptoms in patients with ANOCA.

Regarding to diagnostics, many questions are still open, of which we will discuss a number. Firstly, the diagnostic value of standard non-invasive ischemia detection tests like exercise tests, SPECT, PET, CMR for coronary vascular dysfunction needs to be established as long as invasive coronary vasomotor tests are scarce. Secondly, it would be worthwhile to improve non-invasive diagnostics, especially for coronary vasospasm, which is the most common form of coronary vascular dysfunction. Thirdly, invasive methods to diagnose CMD could be improved. Current methods comprise CFR and IMR measurements using Doppler flow or thermodilution velocity methods which are subject to technical challenges leading to reduced quality measurements and relatively high inter- and intra-observer variability (56). Furthermore, these methods require hyperemia for which intravenous adenosine is administered which can cause side effects including chest pain, dyspnea and AV-blocks. Moreover, it should be avoided in patients with severe chronic obstructive pulmonary disease (COPD) and is contraindicated in patients with asthma.

Recently, a novel method has been validated that allows this direct quantification of absolute coronary blood flow (Q) and resistance (R) using continuous thermodilution (106–108). This method has less technical disadvantages and does not require the use of adenosine. Studies have shown this method to be feasible and safe (107, 109), and Q and R to be related to symptoms (110). Future research is needed to further explore the diagnostic value of this promising technique and establish clinically meaningful cut-off values (111).

Regarding therapeutic strategies, large outcome trials on medication for coronary vascular dysfunction are much awaited. Thereby, it is important to investigate the effect of therapy on the different endotypes of coronary vascular dysfunction (macro- or microvascular vasospasm, reduced vasodilatory capacity, increased microvascular resistance) in order to provide patient-tailored therapy. Apart from medication, non-medical therapy is an essential part of the treatment, especially in patients with refractory symptoms. Future research should be focused on cardiac rehabilitation programs tailored to this patient group with emphasis on exercise but also on stress reduction and coping strategies for chronic, often disabling, symptoms.

## Author Contributions

All authors listed have made a substantial, direct and intellectual contribution to the work, and approved it for publication.

## Conflict of Interest

PD has received consultancy fees from Philips and Abbott, and research grants from Philips, Abbott, and AstraZeneca. PD and SE-S have received a research grant from Abbott. The remaining authors declare that the research was conducted in the absence of any commercial or financial relationships that could be construed as a potential conflict of interest.

## Publisher's Note

All claims expressed in this article are solely those of the authors and do not necessarily represent those of their affiliated organizations, or those of the publisher, the editors and the reviewers. Any product that may be evaluated in this article, or claim that may be made by its manufacturer, is not guaranteed or endorsed by the publisher.
